# Epithelial Cells Are Active Participants in Vocal Fold Wound Healing: An *In Vivo* Animal Model of Injury

**DOI:** 10.1371/journal.pone.0115389

**Published:** 2014-12-16

**Authors:** Ciara Leydon, Mitsuyoshi Imaizumi, Rebecca S. Bartlett, Sarah F. Wang, Susan L. Thibeault

**Affiliations:** Division of Otolaryngology – Head and Neck Surgery, Department of Surgery, University of Wisconsin – Madison, Madison, Wisconsin, United States of America; University of Munich, Germany

## Abstract

Vocal fold epithelial cells likely play an important, yet currently poorly defined, role in healing following injury, irritation and inflammation. In the present study, we sought to identify a possible role for growth factors, epidermal growth factor (EGF) and transforming growth factor-beta 1 (TGFβ1), in epithelial regeneration during wound healing as a necessary first step for uncovering potential signaling mechanisms of vocal fold wound repair and remodeling. Using a rat model, we created unilateral vocal fold injuries and examined the timeline for epithelial healing and regeneration during early and late stages of wound healing using immunohistochemistry (IHC). We observed time-dependent secretion of the proliferation marker, ki67, growth factors EGF and TGFβ1, as well as activation of the EGF receptor (EGFR), in regenerating epithelium during the acute phase of injury. Ki67, growth factor, and EGFR expression peaked at day 3 post-injury. Presence of cytoplasmic and intercellular EGF and TGFβ1 staining occurred up to 5 days post-injury, consistent with a role for epithelial cells in synthesizing and secreting these growth factors. To confirm that epithelial cells contributed to the cytokine secretion, we examined epithelial cell growth factor secretion *in vitro* using polymerase chain reaction (PCR). Cultured pig vocal fold epithelial cells expressed both EGF and TGFβ1. Our *in vivo* and *in vitro* findings indicate that epithelial cells are active participants in the wound healing process. The exact mechanisms underlying their roles in autocrine and paracrine signaling guiding wound healing await study in a controlled, in vitro environment.

## Introduction

Vocal folds are covered by a specialized, multilayered epithelium which protects the underlying tissue from environmental and mechanical insults. To maintain an intact epithelium, epithelial cells undergo constant turnover across the lifespan. Under homeostatic conditions, epithelial renewal is likely achieved by turnover of a small number of cells in the basal layer of the vocal fold epithelium [1]. The cellular and molecular mechanisms underlying epithelial self-renewal in response to daily challenges, injury, and infection are unknown. One common way to elucidate these mechanisms is to recruit cells into a proliferative state through controlled injury. In the present study, we sought to demonstrate the involvement of key growth factors in epithelial regeneration during the acute phase of wound healing as a necessary first step for uncovering potential mechanisms of wound repair and remodeling.

Various growth factors have been implicated in the regeneration of a structurally and functionally intact epithelium after injury. Here, we focus on two endogenous growth factors that are known to play key roles in epithelial proliferation throughout the body, epidermal growth factor (EGF) and transforming growth factor beta (TGFβ1). EGF is secreted by various cell types involved in wound healing including epithelial cells [2], [3], fibroblasts, macrophages, and platelets [4]. The effects of exogenous EGF on vocal fold fibroblast, but not epithelial cell, behavior has been studied *in vitro*; EGF stimulates canine fibroblast proliferation [5], but reduces porcine fibroblast migration in *in vitro* scratch assays [6]. Endogenous EGF has been shown to play a critical role in guiding acute [7] and chronic [8] epithelial response to damage through paracrine and autocrine signaling in airway epithelia. Further, it promotes epithelial regeneration by regulating intercellular junction disassembly and increasing cell proliferation after injury [9], [10]. Determining the presence, expression level, and role of endogenous EGF in epithelium and lamina propria during acute and chronic phases of vocal fold wound healing *in vivo* awaits investigation.

The role of TGFβ has been better explored in vocal fold wound healing; however, its effects are not fully understood. TGFβ is secreted by epithelial cells, fibroblasts, macrophages, and platelets [4]. It is purported to mediate effects that both help and hinder normal wound healing; for example, TGFβ reduces airway epithelial proliferation *in vitro* [11] but stimulates mucosal remodeling (e.g. extracellular matrix (ECM) deposition) *in vivo* in its role as a profibrogenic factor [8]. TGFβ has three isoforms, TGFβ1, TGFβ2 and TGFβ3. Welham and colleagues [12] reported that TGFβ1 and TGFβ3 are expressed in injured and uninjured vocal fold mucosa, in cell-specific and time-dependent manners. With regard to epithelium, TGFβ1 is expressed in naïve rat stratified squamous epithelial cells and ciliated pseudocolumnar epithelial cells while TGFβ3 is expressed in primarily in ciliated pseudocolumnar epithelial cells. Following injury, TGFβ1 is observed throughout rat vocal fold mucosa, while TGFβ3 is observed primarily in epithelial cells [12]. TGFβ1 mRNA expression levels have been quantified in rat vocal fold at various time points during the acute and chronic phases of wound injury; an increase in expression levels was reported at one hour [13], 3 days [14], to seven days post-injury [12], [15]. TGFβ1 regulates the lamina propria composition through synthesis of ECM component such as collagen [16], cell proliferation, and cell death [17]. Expression levels in vocal fold lamina propria post-injury have been correlated with histologic changes in the lamina propria during repair; peak TGFβ1 levels correlated with deposition of collagen type I and type III, as well as fibronectin [18]. Exogenous TGFβ1, the most abundant isoform of the TGFβ superfamily, induces collagen secretion [12], [19] and myofibroblast differentiation [12] by vocal fold fibroblasts *in vitro*, indicating a role for the growth factor in scar formation. *In vivo* experiments have shown that exogenous TGFβ1 and TGFβ3 reduce vocal fold scar formation post-injury [12], [20]. While, based on literature in other tissue [4], [8], [11], autocrine and paracrine TGFβ1 signaling in vocal fold epithelium is likely, TGFβ secretion by epithelial cells has yet to be confirmed.

Both EGF and TGFβ mediate their effects through activation of the epidermal growth factor receptor (EGFR), a member of the ErbB family of receptor tyrosine kinases; EGF activates EGFR by binding directly to the receptor while TGFβ1 activates or inhibits EGFR indirectly via a signaling pathway [21]. This activation is critical for wound repair [2], [22], specifically epithelial proliferation and migration. Given the purported effects of EGF and TGFβ1 on epithelium outlined above, examination of the distribution of activated EGFR following injury can yield important insights into mucosal remodeling. Increased EGFR expression has been reported in airway epithelium in chronic disease *in vivo* [7] and following acute injury [23], [24]. Further, EGFR, which are found only on the basolateral membrane of healthy airway epithelial cells and, therefore, are protected from environmental stimuli, are expressed on the apical membrane of these cells following injury thus exposing them to activation by environmental stimuli [8]. This increased expression and altered distribution of EGFR are associated with hyperresponsiveness to environmental stimuli, inflammation, and mucosal remodeling [8]. Similar changes in expression level and distribution of activated EGFR in vocal fold epithelium following injury would support a role for EGFR in mediating vocal fold mucosal remodeling [8]. While increased EGFR expression has been observed in vocal fold squamous cell carcinoma and benign vocal fold disorders [25], [26], EGFR expression in vocal fold epithelium following injury has not been examined. As a necessary first step to uncovering the role of EGFR in various phases of mucosal remodeling, here we examined the density and location of EGFR in injured vocal fold epithelium during the acute phase of wound healing. We anticipated observing EGFR activation during the proliferative and inflammatory phases of mucosal healing.

Based on findings in other airway epithelia, epithelial regeneration following vocal fold injury likely follows three overlapping steps: cell adhesion and migration, proliferation and stratification, and differentiation [27]. While these steps have not been explicitly identified in vocal fold epithelial regeneration, their occurrence can be inferred from literature. Cell adhesion and migration, as evidenced by an emerging but incomplete layer of epithelium, will occur three days post-injury [28]–[31]. Epithelial cell proliferation and stratification will occur five days post-injury. We hypothesized that these migratory and proliferative stages of repair will be marked by heightened levels of expression of ki67, a maker of proliferation, K14, a marker of proliferative epithelial cell, EGF, TGFβ1 and EGFR. A confluent, multilayered epithelium will be present within 14 days post-injury [28], [31]. Cell proliferation will be expected to continue to be elevated until 14 days post-injury. We anticipatedthat this stage will be marked by elevated Ki67 and K14 expression. Based on observations of cell differentiation in airway epithelia [32], we anticipate that a fully differentiated, complete epithelium will be restored within 35 days following injury. At day 35, Ki67 will be expected to return to uninjured levels, consistent with return of a complete, stable epithelium. Further, observation of K14 staining in the basal layer only, will be consistent with non-injured, differentiated stratified epithelium. Although the studies cited above provide a likely timeline of the critical stages of epithelial regeneration, there remain important gaps in our knowledge of the re-epithelialization process. First, we needed to establish a timeline for regeneration of an epithelium. Second, we needed to demonstrate the presence of growth factors in the epithelium likely to play an important role in each stage of epithelial regeneration. Consequently, we elected to examine wound healing in a rat model at five time points (1, 3, 5, 14 and 35 days) post-injury. These time points were chosen to determine the sequence and timing of each stage of epithelial healing. Third, we needed to confirm that epithelial cells, not a different cell type, secreted observed growth factors. To that end, we examined EGF and TGFβ1 expression in primary epithelial cells. Given the low number of epithelial cells in a rat vocal fold and the difficulty in isolating rat epithelial cells from the underlying lamina propria because of its small size, quantification of gene and protein expression by epithelial cells was not feasible. Therefore, we used primary pig cells to confirm EGF and TGFβ1 gene expression in vocal fold epithelial cells.

## Materials and Methods

### Animals

Fifteen three-month-old male Sprague Dawley rats were used in this study. The animal protocol was implemented with prior approval from the School of Medicine and Public Health Institutional Animal Care and Use Committee of the University of Wisconsin-Madison (Permit number: M02459). All animals underwent unilateral vocal fold surgery. The surgical procedure has been described previously elsewhere [33]. Briefly, rats were anesthetized with isoflurane (2% to 3% delivered at 0.8 to 1.5 L/min) followed by an injection of xylazine (9 mg/kg) and ketamine (90 mg/kg), and placed in a near-vertical position on a custom designed stand. The mouth was secured in an open position using a custom-made device [34]. Vocal folds were visualized with a 1.9 mm 30-degree endoscope. Using a 25-gauge spinal needle, the epithelium and lamina propria of one vocal fold were removed to expose the underlying thyroarytenoid muscle. Three rats were sacrificed by exposure to carbon dioxide at each of five time points (1, 3, 5, 14 and 35 days) post-surgery. Larynges were harvested immediately following euthanasia and processed for routine histology and immunohistochemistry.

### Histology and Immunohistochemistry

Larynges were fixed in 10% neutral phosphate-buffered formalin (pH of 7.0) overnight at room temperature, processed, and embedded in paraffin. Serial coronal sections, 5 µm thick, were cut along the length of the membranous vocal folds. Routine hematoxylin and eosin (H&E) staining was performed on sections at 100 µm increments. Standard immunohistochemistry was completed. Antigen retrieval was achieved using heat-activated 10 mM sodium citrate buffer (pH 6). Sections were incubated for one hour at room temperature using antibodies directed against the proliferation marker, Ki67 (1∶100; ab16667, Abcam); transforming growth factor beta 1 (TGFβ1, 1∶100; sc-52983, Santa-Cruz Biotechnology); epidermal growth factor (EGF, 1: 1∶100; BT-585, Biomedical Technologies Inc.); phosphorylated epidermal growth factor receptor (Tyr1068, 1∶100; 3777, Cell Signaling Technology); and keratin 14, a marker of basal layer cells (K14, 1∶200; RB-9020, Thermo Scientific). Species-specific isotype controls were used as negative controls. Endogenous peroxidase activity was quenched by incubation of sections in 3% hydrogen peroxide. For anti-EGF and anti-TGFβ1 antibodies, sections were treated with a goat anti-rabbit biotinylated secondary antibody (1∶200; Vector Labs) followed by incubation in a universal prediluted streptavidin conjugated to horseradish peroxidase (Vector Labs). For the remaining antibodies, sections were treated with goat anti-rabbit prediluted Impress secondary antibodies (Vector Labs). All sections were visualized with a diaminobenzidine (DAB) chromagen and counterstained with hematoxylin. They were viewed using a Nikon E600 microscope and photographed using an Olympus DP71 microscope digital camera.

### Cell culture

Primary epithelial cells were derived from pig larynges obtained from an abattoir (Black Earth Meats, Black Earth, WI) using a protocol described elsewhere [35]. Briefly, vocal folds were washed with sterile Hanks' Balanced Salt Solution (HBSS) and soaked overnight in an incubator at 37°C in dispase I (1 U/ml; Roche) to separate the epithelium from the underlying lamina propria. Sheets of epithelium were placed in trypsin-EDTA (Life Technologies) for 10 minutes to isolate cells, then vortexed and submerged in soybean trypsin inhibitor (Sigma). The cell suspension was filtered through a cell strainer (40 µM) and centrifuged at 188 g for 5 minutes at 4°C. Supranatant was removed and cells were resuspended in flavinoid adenine dinucleotide (FAD) media. The media was composed of Ham's F-12/DMEM (3:1 ratio), FBS (2.5%), hydrocortisone (0.4 µg/ml), cholera toxin (8.4 ng/ml), insulin (5 µg/ml), adenine (24 µg/ml), epidermal growth factor (10 ng/ml), penicillin (100 U/ml) and streptomycin (0.01 mg/ml). Cells were plated in tissue culture treated six-well plates, submerged in FAD, placed in a 37°C incubator with 5% carbon dioxide and grown to confluence. They were harvested for PCR analysis at five to seven days after plating.

### Polymerase chain reaction

Pig epithelial cells were grown to approximately 80% confluency before being harvested for PCR. Messenger RNA from was obtained using standard methods. Total RNA was isolated using an RNA extraction kit (RNeasy Mini Kit; Qiagen). First strand cDNA was generated from 2 µg of total RNA using a QuantiTect Reverse Transcription Kit (Qiagen). Primer pairs reported elsewhere were used for EGF [36], TGF β1 [37], and beta-actin (Table 1). The latter served as a control. An annealing temperature of 55°C was used for EGF and β-actin, and 56°C for TGFβ1. Products were run on a 1% agarose gel and imaged after incubation with an ethidium bromide solution using a BioDoc-It Imaging System (UVP).

**Table 1:** Primer sequences and products for polymerase chain reaction (PCR).

Gene	Forward Primer	Reverse Primer	Size
**TGFβ1**	CGATTAAAGGTGGAGAGAGGACTG	AATGAATGGTGGACAGACACAGG	128 bp
**EGF**	TCTGAACCCGGACGGATTTG	GACATCGCTCGCCAACGTAG	202 bp
**β-actin**	ACGTTGCTATCCAGGCTGTGCTAT	CTCGGTGAGGATCTTCATGAGGTAGT	188 bp

## Results

**Figure 1:**
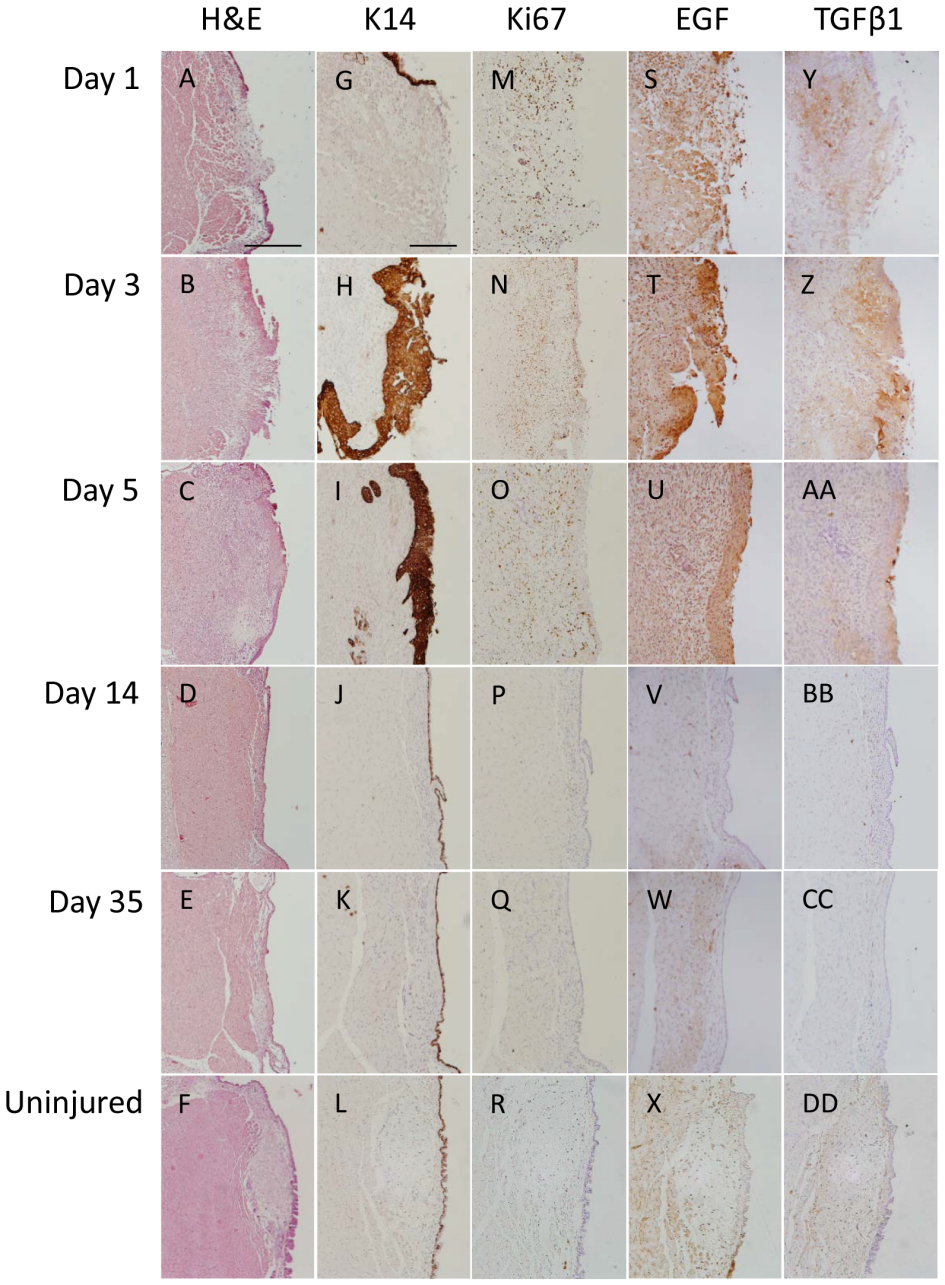
Epithelial regeneration following vocal fold injury. Hematoxylin and eosin (H&E) in injured vocal folds from day 1 (D1) to day 35 (D35) post-injury and uninjured vocal folds (A–F). Immunostaining (brown) for keratin 14 (K14; G–L); Ki67 (M–R); epidermal growth factor (S–X); and transforming growth factor beta (TGFβ1) at five time points ranging from 1 to 35 days post-injury, as well as in uninjured vocal folds (Y-DD). Images show results for representative animals. Scale bar: 100 µm.

In the present study, the vocal fold epithelium and lamina propria were removed, exposing the underlying thyroarytenoid (TA) muscle. Injury to the depth of the TA was confirmed by observation of disorganization of the most medial muscle fibers and infiltration of cells around these fiber bundles one day post-injury (Fig. 1A). Epithelium was absent at day 1 post-injury. By day 3, most of the wound was covered by epithelium; however, epithelium remained absent in small areas at this time point (Fig. 1B). At day 5, epithelial hyperproliferation was observed (Fig. 1C). By day 14, a thin epithelium characteristic of uninjured rat vocal folds was restored (Fig. 1D, 1F).

The epithelial cells demonstrated positive staining for K14, a marker of basal cells in uninjured epithelium, throughout all epithelial layers at days 3 and 5 post-injury (Fig. 1H,I). By day 14, K14 was found primarily in basal layer of the epithelium (Fig. 1J). This pattern of staining was also seen at 35 days post-injury and in uninjured vocal folds (Fig. 1K, 1L).

Positive staining for Ki67, a marker of cell proliferation, was found primarily in the lamina propria at day 1 post-injury (Fig. 1M). While epithelium was absent at the wound site at that time point, some staining was noted in the at a distance of around 10 cells from the wound margins at that time point; however, no staining was found in the epithelium immediately adjacent to the wounded area consistent with a lag in healing immediately post-injury. Abundant staining was observed in all epithelial cell layers at day 3 (Fig. 1N). By day 5, heterogeneous distribution was no longer observed; staining was confined primarily to the basal layer of the hyperplastic epithelium (Fig. 1O). Strong cell staining was also noted in the lamina propria through day 5 post-injury. By day 14, Ki67 was absent from the epithelium and lamina propria (Fig. 1P). Ki67 staining was not observed at 35 days post-injury or in uninjured vocal folds (Fig. 1Q, 1R).

**Figure 2:**
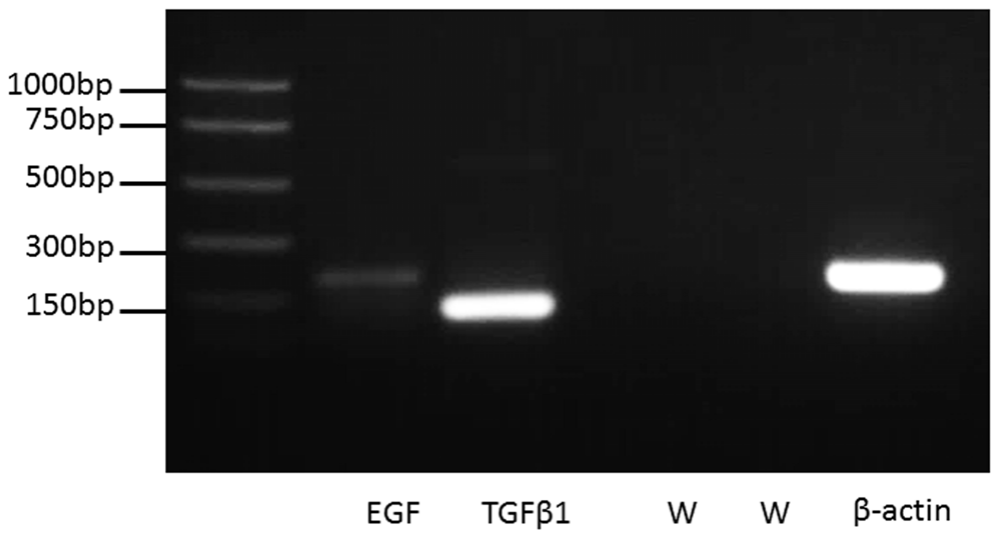
Growth factor expression in primary pig vocal fold epithelial cells. Agarose gel electrophoresis of PCR products shows expression of EGF (lane one), TGFβ1 (lane two), and the positive control (lane five) in primary pig vocal fold epithelial cells at the expected product sizes. Negative water controls (W) for EGF and TGFβ1 are shown in lanes three and four, respectively.

At day 1, EGF was found throughout the lamina propria, both associated with cells and in the extracellular matrix (Fig. 1S; S1A Figure). By day 3, cytoplasmic and intercellular staining was observed in all layers, with strongest staining noted in the most superficial layers of the epithelium and near erythrocytes within the epithelium (Fig. 1T; S1B Figure). By day 5, the stain was noted predominately in the most superficial layers of epithelial cells with intense staining around blood cells (Fig. 1U; S1C Figure shows greater detail). EGF staining was absent in the epithelium and sparse in the lamina propria at days 14 and 35 post-injury (Fig 1V, 1W), and in uninjured vocal folds (Fig. 1X). EGF expression in epithelial cells was confirmed via PCR (Fig. 2).

Staining for TGFβ1 was noted in the basal layer of the epithelium at the wound margins, adjacent to the site of injury, and throughout the lamina propria at day 1 post-injury (Fig. 1Y; S2A Figure). By day 3, cytoplasmic and intercellular staining was present throughout the epithelium but remained strongest at the interface between the epithelium and lamina propria (Fig. 1Z; S2B Figure). By day 5, as with EGF, the staining was noted predominately in the most superficial layers of epithelial cells (Fig. 1AA; S2C Figure). TGFβ1 staining was largely absent at days 14 and 35 post-injury (Fig. 1BB, 1CC), and from uninjured vocal folds (Fig. 1DD). Expression of TGFβ1 in epithelial cells was confirmed using PCR (Fig. 2).

**Figure 3:**
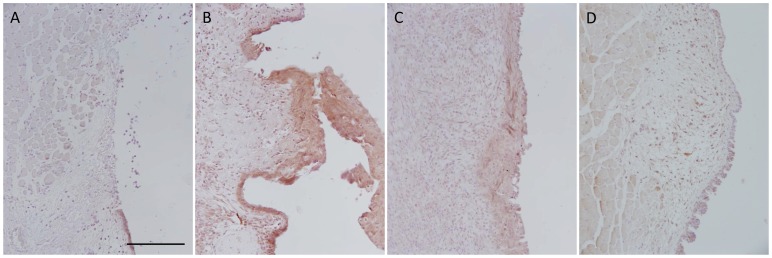
EGFR expression during the acute phase of vocal fold wound healing. Positive immunostaining (brown) for activated EGFR was present in epithelium around the wound margin at day 1 post-injury (A). Staining was diffuse and abundant at day 3 post-injury (B). Staining was observed in discrete cells at day 5 post-injury (C). Staining was absent from uninjured epithelium (D). Images show results for representative animals. Scale bar: 100 µm.

EGFR activation was noted in epithelial cells adjacent to the site of injury one day post-injury (Fig. 3A). Abundant staining in all epithelial cell layers was observed at day 3 (Fig. 3B). By day 5, staining was restricted primarily to cells in the basal and most superficial layers of epithelial cells (Fig. 3C). Staining was largely absent from the epithelium of uninjured vocal folds (Fig. 3D).

## Discussion

Vocal fold healing post-injury is likely dictated by the interactions between various cells types, growth factors and components of the extracellular matrix. Here, we focused on establishing a timeline for epithelial restoration and presence of two growth factors likely to play an important role in wound healing in a rat model. Through histological and immunohistological assays, we demonstrated that epithelium follows the expected temporospatial sequence of wound healing observed in other airway epithelia. We further demonstrated that epithelial cells are active participants in the wound healing process as evidenced by secretion of growth factors critical for wound healing, EGF and TGFβ1, as well as activation of EGFR.

Based on findings in other airway epithelia and studies of vocal fold mucosal repair, we hypothesized that epithelial regeneration following vocal fold injury would follow three steps: cell adhesion and migration, proliferation and stratification, and differentiation [27]. Our findings were consistent with the predicted sequence and timeline in epithelial healing described above. Cell adhesion and migration, as evidenced by an emerging but incomplete layer of epithelium viewed with H&E, occurred three days post-injury. By day five, a fully confluent, multilayered epithelium was observed. Epithelial regeneration was evaluated by staining for Ki67, a marker of cell proliferation. Epithelial proliferation was initiated one day post-injury. However, proliferation was sparse and noted in the epithelium up to ten cells in distance from the wound one day post-injury consistent with a lag period in healing immediately post-injury. This lag period has been observed in various tissues immediately after injury and is attributed to cellular reorganization and protein synthesis prior to cell proliferation and migration [22]. Epithelial cell proliferation peaked at 3 days and remained elevated at 5 days post-injury. By day 14, proliferation levels returned to pre-injury levels as evidenced by sparse to absent Ki67 staining in the epithelium. Interestingly, at early time points post-injury, cells throughout the epithelium, not just in the basal layers, stained positive for ki67. This indicates that cells other than the basal cells, which drive epithelial proliferation under homeostatic condition, are recruited to proliferate post-injury. Further, K14 positive staining, which is typically restricted to the basal layer, was observed throughout the epithelium at days 3 and 5 post-injury. Together, these findings suggest that epithelial cells in suprabasal layers were capable of cell division post-injury suggesting that terminal differentiation of cells in the suprabasal layers had not occurred by day 5. A typically differentiated epithelium was restored by 14 days following injury, as evidenced by an absence of K14 staining in suprabasal cells of the two-layered epithelium.

Epithelial cells showed positive staining for the growth factors, EGF and TGFβ1 during the early phase of wound healing. Further, the cytoplasmic and intercellular staining observed for both EGF and TGFβ1 is consistent with a role for the epithelial cells in synthesizing and secreting these growth factors. These findings suggest that vocal fold epithelial cells may participate in autocrine and paracrine signaling in wound healing. Presence of positive staining for growth factors in epithelium during the proliferation and migration phases of epithelial regeneration post-injury strongly support our hypothesis that epithelial cells participate in all phases of epithelial regeneration through autocrine signaling. EGF and TGFβ1 showed a similar, time-dependent pattern of staining across early time points post-injury. Both showed diffuse staining throughout the epithelium at day 3 and strongest staining in the superficial layer of the epithelium at day 5. Presence of growth factors in the superficial epithelial layers in the days following injury is consistent with a role for them in guiding proliferation and differentiation of these metabolically active but typically quiescent cells [1]. The possibility that these cells are recruited to proliferate following injury is supported by the absence of terminal differentiation of the cells (as evidence by K14-positive staining) and staining for the proliferation marker, Ki67 in these cells. The presence of red blood cells in the superficial layer of the epithelium is important in the context of this study as they provide additional sources of growth factors. The sparse, if not absent staining, for the growth factors at later time points suggests that TGFβ1 and EGF are not secreted by epithelial cells under homeostatic conditions following restoration of a complete, differentiated epithelium.

EGFR activation was observed during the acute phase of wound healing. Concomitant staining for EGFR and the growth factors, EGF and TGFβ1, in epithelial cells suggest growth factor-mediated autocrine signaling drives EGFR regulation of wound repair. Presence of activated EGFR in the proliferating, Ki-67-positive, epithelium is consistent with the receptor playing a key role in cell growth and wound repair. Our findings are consistent with a correlation between EGFR and Ki67 expression observed in benign and malignant laryngeal lesions [25].

Growth factors were also noted in the lamina propria, particularly during the earliest time points post-injury, day 1 and 3. These observations were expected as both EGF and TGFβ1 are secreted by fibroblasts, macrophages, and platelets [4], all of which are present during the early phases of wound healing. Observation of TGFβ1 staining in the ECM of the lamina propria is consistent with secretion of TGFβ1 by cells such as fibroblasts [12], and, we hypothesize, storage of TGFβ1 as observed in other ECM [38]. Further, increased TGFβ gene expression has been observed during the first week post-injury in rats [12]–[15]. To the best of our knowledge, EGF gene and protein expression have not be quantified in a rat model of vocal fold injury.

We acknowledge two weaknesses in this study. First, dynamic reciprocity between epithelium and lamina propria likely molds vocal fold healing. Here, we focused exclusively examining on epithelial cell protein expression as a first step to elucidating the role of epithelial cells in mediating wound healing. Understanding the interaction of epithelial cells and fibroblasts, as well as other cells, in the lamina propria awaits controlled, in vitro studies of vocal fold healing. Second, we did not quantify EGF and TGFβ1 gene and protein expression in rats post-injury. We used immunohistochemistry (IHC) for the in vivo portion of the study. While IHC did not permit quantification of protein expression, it allowed visualization of the cells that expressed EGF and TGFβ1. TGFβ gene expression has been quantified during the acute and chronic phases of wound healing in vocal fold mucosa [12]–[15]. However, it is unknown which cells contributed to the aggregate cytokine secretion; while vocal fold mucosa contains epithelial cells, it also contains many other cell types including fibroblasts, macrophages, and platelets, each capable of secreting growth factors. Our interest was to examine growth factor secretion by epithelial cells. We showed that vocal fold epithelial cells do express TGFβ1 and EGF. Our observation of a positive, albeit faint, EGF band was consistent with low expression levels of EGF in other stratified squamous epithelia, including corneal epithelium [39] and bronchial epithelium [40]. While it would have been ideal to demonstrate gene and protein expression in the same species, the absence of primary rat epithelial cell rendered this infeasible. Instead, we used primary pig cells which have been characterized elsewhere [35].

To date, studies have focused on analyzing changes in composition and organization of the lamina propria and their impact on vocal fold physiology following damage, with few exceptions [12], [30]. Examining the cellular and extracellular composition and biomechanical properties of the lamina propria is important as they determine key clinical parameters such as vocal quality and ease of phonation. However, lack of appreciation of the cellular and molecular changes underlying epithelial regeneration and restoration of epithelial barrier function following vocal fold injury results in an incomplete understanding of wound healing. The role of epithelium in modulating vocal fold wound healing and the mechanisms underlying epithelial regeneration had not been examined even though an intact epithelium is critical for protecting the vocal folds from injury, as well as maintaining vocal fold defense [41] and surface ion and fluid homeostasis [42]. Here we show that epithelial cells are active participants in the healing process. The exact mechanisms and significance of the EGF and TGFβ1-mediated inhibitory and excitatory pathways guiding wound healing in vocal fold await study in a controlled, *in vitro* environment. Knowledge of these mechanisms of epithelial wound healing may help to inform development of growth factor driven interventions over time. It may also provide a benchmark against which the ability of in vitro models of vocal fold injury under development to mimic in vivo injury [43] can be evaluated.

## Supporting Information

S1 Figure **EGF staining in the acute phase of healing.** Positive EGF staining (brown) was observed in epithelium at 1 (A), 3 (B), and 5 (C) days post-injury in representative animals. Scale bars: 50 and 100 µm.
(TIF)

S2 Figure **TGFβ1 staining in the acute phase of healing.** Positive TGFβ1 staining (brown) was observed in epithelium at 1 (A), 3 (B), and 5 (C) days post-injury in representative animals. Scale bars: 50 (insert) and 100 µm.
(TIF)
