# Supplementary figures and images for: Epithelial Cells Are Active Participants in Vocal Fold Wound Healing: An In Vivo Animal Model of Injury
Source: PLoS One. 2014 Dec 16;9(12):e115389. doi: 10.1371/journal.pone.0115389 (PMC4267843; doi:10.1371/journal.pone.0115389)

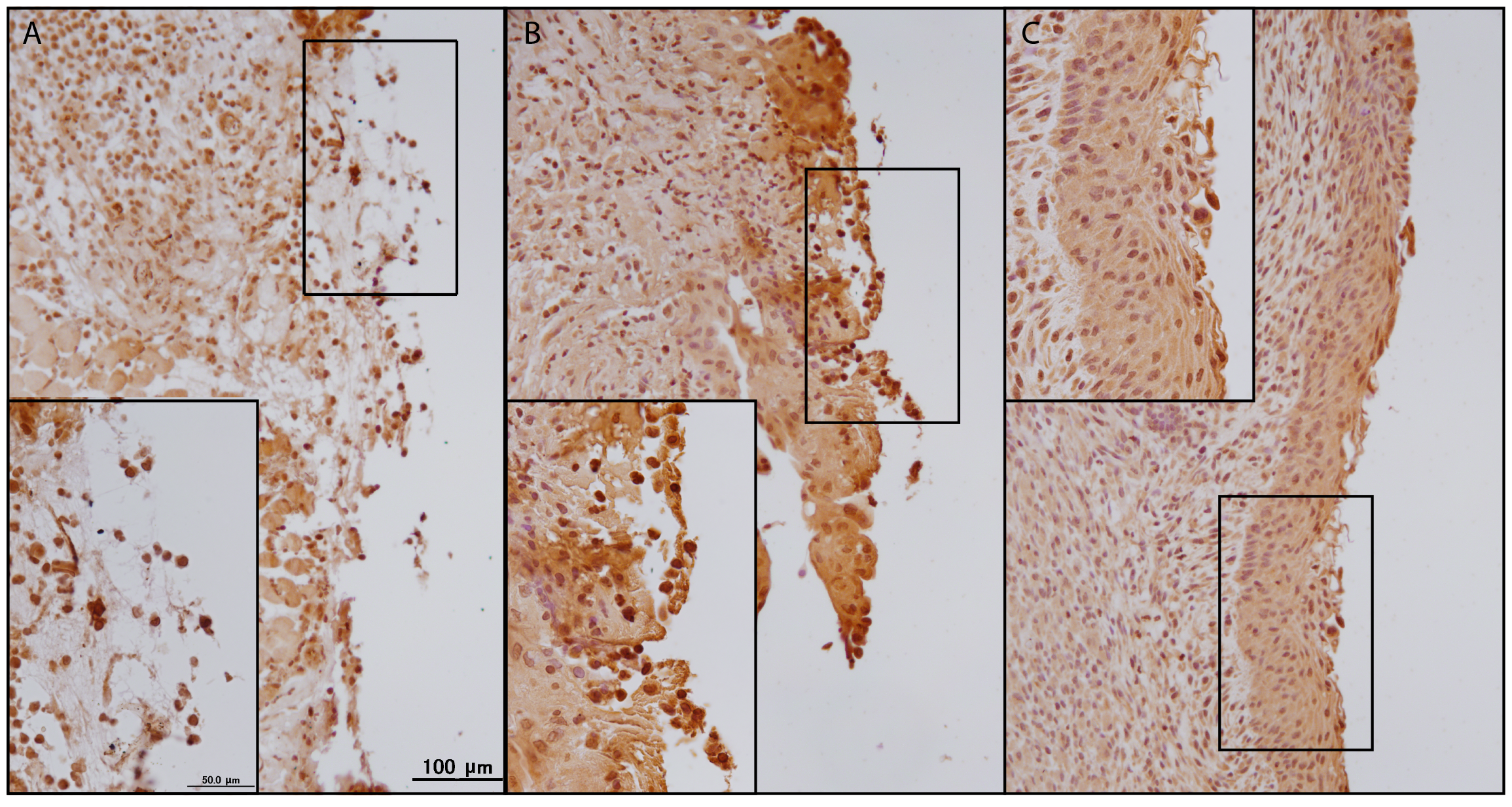

Supplement: S1 Figure — EGF staining in the acute phase of healing. Positive EGF staining (brown) was observed in epithelium at 1 (A), 3 (B), and 5 (C) days post-injury in representative animals. Scale bars: 50 and 100 µm. (TIF) [file pone.0115389.s001.tif]

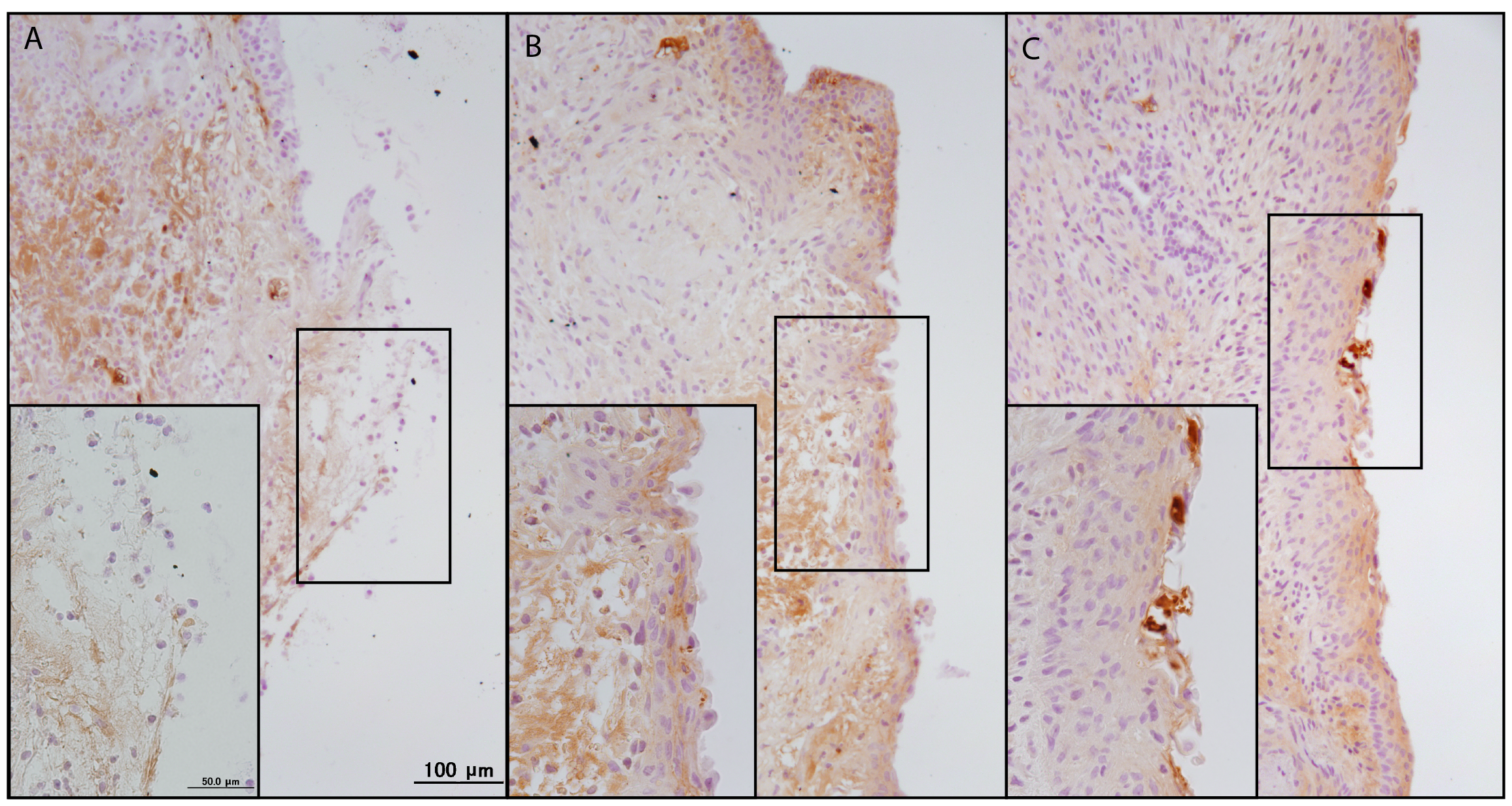

Supplement: S2 Figure — TGFβ1 staining in the acute phase of healing. Positive TGFβ1 staining (brown) was observed in epithelium at 1 (A), 3 (B), and 5 (C) days post-injury in representative animals. Scale bars: 50 (insert) and 100 µm. (TIF) [file pone.0115389.s002.tif]
